# CD73 Activity is Dispensable for the Polarization of M2 Macrophages

**DOI:** 10.1371/journal.pone.0134721

**Published:** 2015-08-10

**Authors:** Dominik Eichin, Juha P. Laurila, Sirpa Jalkanen, Marko Salmi

**Affiliations:** 1 Medicity Research Laboratory, University of Turku, Turku, Finland; 2 Department of Medical Microbiology and Immunology, University of Turku, Turku, Finland; University of Colorado Denver, UNITED STATES

## Abstract

The ectoenzyme CD73 catalyzes the hydrolysis of AMP, and is one of the most important producers of extracellular adenosine. On regulatory T cells, CD73 is necessary for immunosuppressive functions, and on Th17 cells CD73-generated adenosine exerts anti-inflammatory effects. However, the expression and function of CD73 in pro-inflammatory M1 and in immunosuppressive M2 macrophages is largely unknown. Here we show that CD73 expression and enzyme activity were induced in *in vitro* polarized pro-inflammatory human M(LPS+TNF) monocytes/macrophages, while CD73 was absent from immunosuppressive M(IL-4+M-CSF)-polarized macrophages. Inhibition of CD73 activity with the inhibitor AMPCP did not affect the polarization of human monocytes. In mice, CD73 was present on resident peritoneal macrophages. In striking contrast, elicited peritoneal macrophages remained CD73 negative regardless of their polarization towards either a pro-inflammatory M(LPS) or anti-inflammatory M(IL-4c) direction. Finally, the ability of peritoneal macrophages to polarize to pro- and anti-inflammatory cells was perfectly normal in CD73-deficient mice *in vivo*. These data indicate that, in contrast to other major leukocyte subpopulations, CD73 activity on macrophages does not play a major role in their polarization and that in mice host CD73 on any cell type is not required *in vivo* for peritoneal macrophage polarization towards either a pro- or an anti-inflammatory direction.

## Introduction

CD73 (ecto-5’-nucleotidase) is an ectoenzyme expressed, among other cell types, on dendritic cells as well as on a subset of B- and T-lymphocytes, including regulatory T cells (Tregs) [1,2]. It is a key control element in regulating the magnitude and duration of extracellular purinergic signaling [3]. In this cascade, CD39 dephosphorylates pro-inflammatory ATP to ADP and AMP whereupon CD73 hydrolyses AMP to adenosine. Binding of adenosine to one of the four G-protein coupled adenosine receptors triggers several anti-inflammatory and immune-suppressing functions in the immune system [4,5].

In mice, Tregs produce adenosine in a CD73-dependent manner [6]. Adenosine promotes Treg expansion and triggering of adenosine receptor 2A on effector T-cells induces NF-kB mediated suppression of cytokine production as well as other anti-inflammatory effects. The immunosuppressive functions of human Tregs also require CD73-mediated adenosine production [7]. In *in vitro* studies, Tregs induce the polarization of macrophages towards an immunosuppressive phenotype both in mice and humans [8,9]. Moreover, in Th17 cells induction of CD73 (and CD39) is needed for immunosuppressive functions [10], and in neutrophils, adenosine generation through CD73 activity leads to feedback inhibition [11]. Furthermore, CD73 activity decreases adhesion of leukocytes to endothelial cells. These data are in line with observations that *in vivo* CD73-deficient mice develop aggravated inflammatory reactions, for example in inflammatory bowel disease, glomerulonephritis, atheroma and tumor models [1–3].

The role of CD73 within the heterogeneous group of macrophages has not been studied thoroughly [12]. Tissue macrophages can originate from embryonic and hematopoietic origins and are able to adopt multiple different polarization programs within the tissues [13–18].

This divides them into a wide range of phenotypes ranging from pro-inflammatory macrophages on the one side, to anti-inflammatory and tissue regenerative macrophages on the other. While these cells are commonly called M1 or M2 macrophages, respectively, this nomenclature is not precise and recently a unified nomenclature highlighting the nature of the polarizing stimuli has been proposed [19].

Macrophages are polarized into the pro-inflammatory direction by bacterial products or cytokines such as lipopolysaccharide (LPS), IFN-gamma and TNF, whereas they polarized towards an anti-inflammatory direction after exposure to IL-4, IL-10, IL-13, immune complexes or M-CSF [20]. Interestingly, adenosine has been reported to reduce the production of pro-inflammatory mediators and increase the production of anti-inflammatory mediators in macrophages. [5,21,22]. Notably, the polarization pathways are highly dynamic and flexible, consisting of multiple intermediate states and subtypes.

Normally most circulating monocytes, with the exception of a minor CD14+CD16+ population, do not show any expression of CD73, neither in human nor in mouse [23,24]. However, about 30% of monocytes and macrophages retrieved from the synovial fluid of arthritic mice do so [25], indicating that under certain circumstances CD73 can be induced also on macrophages. Furthermore, in CD73-deficient mice augmented tumor immunity has been linked to decreased numbers of intratumoral anti-inflammatory macrophages [26], suggesting that CD73 could control macrophage polarization. In fact, recent studies propose that *in vitro* induction of peritoneal mouse macrophages with LPS decreases CD73 expression, while induction with IL-4 increases it [12,27]. Therefore, our aim was to analyze which types of human and mouse macrophages express CD73 and whether CD73 activity, like in other leukocyte subtypes, is required for polarization of immunosuppressive monocytes/macrophages *in vivo*.

## Materials and Methods

### Extraction of human monocytes and polarization

Human blood samples were collected into six Venosafe-EDTA(K_2_) evacuated blood collection tubes each (Terumo, Gothenburg, Sweden) from multiple individuals belonging to the research group after obtaining an oral informed consent from each donor. No information was collected from the donors. The samples were anonymized during the separation of the cells. Thus, the blood was drawn in the absence of the researcher by a trained laboratory nurse, who also separated the mononuclear cells before giving them in an anonymized way to the researcher for the experimentation. All steps of sample preparation and analysis were thus conducted in a manner that ensured that any information derived from an individual sample could not be linked to an individual donor. Verbal informed consent was given to and documented by the researcher in charge as well as the qualified blood-sampling laboratory nurse. The regional ethics committee was not consulted prior to the study, as the procedure of anonymous blood sample collection and analysis, with no personal information collected from the donors, is not considered to be regulated by Finland’s Medical Research Act No. 499/1999.

Monocytes were extracted form peripheral blood by Ficoll-Paque PLUS (GE Healthcare, Helsinki, Finland) followed by negative MACS beads selection (Monocyte Isolation Kit II; MiltenyiBiotec, Lund, Sweden) of CD14^+^CD16^-^ cells according to the manufacturer’s instructions. Monocytes were then cultured in medium (RPMI 1640 supplemented with 10% FCS, 4 mM L-glutamine, 100 U/mL penicillin and 100 μg/mL streptomycin) and polarized to M(LPS+TNF) cells (0.05 U/μL TNF and 1 μg/mL LPS) or to M(IL-4+M-CSF) cells (10 ng/mL IL-4 and 10 ng/mL hM-CSF) for 3 days. In certain experiments, cells were polarized with LPS (1μg/mL) alone or with LPS and IFN-γ (1 μg/mL LPS, 25 ng/mL IFN-γ) (all polarizing molecules except IFN-γ were from R&D Systems, Abingdon, United Kingdom; Recombinant Human IFN-γ was from Peprotech, London, United Kingdom).

### Enzymatic assays

Enzymatic activities of extracted/polarized human cells were determined by using [2-^3^H]AMP (Amersham Biosciences/GE Healthcare, Uppsala, Sweden), [2,8-^3^H]ADP (PerkinElmer, Turku, Finland) and [2,8-^3^H]ATP (PerkinElmer, Turku, Finland) as tracer substrates, as described [28]. Briefly, to all reactions 4 mM β-glyerophosphate (Sigma, Helsinki, Finland) was added and adenosine triphosphatase (ATPase) and adenosine diphosphatase (ADPase) activity was measured by incubating 2*10^4^ cells with 300 μM [2,8-^3^H]ATP or 300 μM [2,8-^3^H]ADP together with 100 μM diadenosine pentaphosphate (Ap_5_A; Sigma, Helsinki, Finland), respectively, for 1 hour at 37°C. Ecto-5’-nucleotidase activity was measured by incubating 5*10^4^ cells with 50 μM [2-^3^H]AMP; adenylate kinase activity by incubating 2*10^4^ cells with 300 μM [2-^3^H]AMP and 700 μM γ-phosphate-donating ATP for 1 hour at 37°C. Incubated aliquots were applied onto Alugram G/UV_254_ plates (Macherey-Nagel, Düren, Germany) and separated by using TLC with 1-Butanol, 3-Methyl-1-butanol, Diethylene glycol monoethyl ether, Ammonia and Milli-Q-Water (9:6:18:9:15) as solvent. Enzymatic activities were measured by scintillation β-counting on a Wallac 1409 (PerkinElmer, Turku, Finland) and expressed as nmol of labelled substrate which was metabolized by one million cells per hour.

### Animals

Specific pathogen free, 2–3 months old male CD73^-/-^mice [29], and respective C57BL/6 wild types were used (Animal license number 3791/04.10.03/2011). They received food and water ad libitum. Animals were bred and housed at the Central Animal Laboratory at the University of Turku, according to international guidelines on the care and use of laboratory animals. All animal experiments were approved by the Finnish Animal Ethics Committee and were performed in compliance with the 3R principle.

### *In vivo* polarization model

M(LPS) polarization was induced by intra-peritoneal (i.p.) injection of 4% thioglycollate on day 0 for 16 h followed by 5 μg LPS (Sigma, Helsinki, Finland) for an additional 6 h before sacrificing the animals. Induction of M(IL-4c) polarization was carried out as described in [30]. Briefly, on day 0 the animals were i.p. injected with a mixture containing 5 μg IL-4 (Peprotech, Stockholm, Sweden) and 25 μg anti-IL4 antibody (BD Biosciences, Vantaa, Finland) in 800 μL 4% thioglycollate. On day 2 the same IL-4-anti-IL-4 antibody complex in 200 μL PBS (Sigma, Helsinki, Finland) was i.p. injected. Animals were sacrificed on day 4. M(-)_LPS_ and M(-)_IL-4c_ denote experimental controls which received the thioglycollate injections but not the subsequent inducers of polarization. Peritoneal cells, containing the polarized monocytes/macrophages, were extracted by flushing the peritoneum with 10 mL RPMI 1640 (Sigma, Helsinki, Finland).

### Flow cytometry

Human cells were pre-blocked with 100 μg/mL human Ig (KIOVIG, Baxter, Helsinki, Finland) and stained with anti-CD73 (mouse IgG2b, in-house, clone 118), anti-MRC1/CD206 (mouse IgG1, #LS-C40886, LifeSpan BioSciences, Helsinki, Finland) and anti-CD14 PB (mouse IgG2a, #558121, BD Biosciences, Vantaa, Finland). Isotype matched negative control antibodies were AK1 (mouse IgG1, in house), mouse IgG2b (Biolegend, #401202, Helsinki, Finland) and mouse IgG2a PB (BD Biosciences, #558118, Vantaa, Finland). Anti-mouse IgG1 AF488 (Invitrogen, #A21121, Stockholm, Sweden) or 1:500 anti-mouse IgG2b PE (SouthernBiotech, #1090–09, Helsinki, Finland) were used as second-stage reagents, as appropriate.

Mouse peritoneal cells were pre-blocked with Fc-block (BD Biosciences, #553142, Vantaa, Finland), before stained for 30 min on ice with the following specific antibodies: anti-F4/80 FITC (rat IgG2b, #MCA497A488), anti-CD206 AF488 (rat IgG2a, #MCA2235A488) (both from AbD Serotec, Puchheim, Germany), anti-Ly6C FITC (rat IgG2c, #128006) and anti-F4/80 (rat IgG2a, #123110) (both from Biolegend, Helsinki, Finland), anti-CD5 PE (rat IgG2a, #553023, BD Pharmingen, Vantaa, Finland), anti-CD11b APC (rat IgG2b, #553312), anti-CD73 PE (rat IgG2a, #550741), anti-Ly6G PE (rat IgG2a, #551461) (all from BD Biosciences, Vantaa, Finland), anti-Ym1 (rabbit polyclonal, #01404, Stemcell Technologies, Grenoble, France) or anti-RELM alpha (rabbit polyclonal, #ab39626, Abcam, Cambridge, UK). Anti-rabbit IgG FITC (Sigma, #F7512, Helsinki, Finland) was used to detect the rabbit polyclonal primary antibodies. Isotype matched negative control antibodies (rat IgG2a AF488 (#400525) and rat IgG2c FITC (#400705) (both from Biolegend, Helsinki, Finland), mouse IgG2a PB (#558118) and rat IgG2b APC (#553991) (both from BD Biosciences, Helsinki, Finland), rat IgG2a PE (#553930) (BD Pharmingen, Helsinki, Finland), rat IgG2b FITC (AbD Serotec, #MCA1125F, Puchheim, Germany)) or purified rabbit Ig were used in all stainings, as appropriate.

Monocytes/macrophages were FSC/SSC gated, followed by gating on CD14^+^ (human cells) or CD11b^+^F4/80^+^ (mouse cells) as shown in S1 Fig. Permeabilization was done with BD Cytofix/Cytoperm (BD Biosciences, Helsinki, Finland). All samples were recorded on a LSRII flow cytometer (BD, Helsinki, Finland) and analyzed using Flowing Software 2.5.0. (BTK, Turku, Finland). Antibodies were used at a concentration of 10 μg/mL or as recommended by the manufacturer, unless otherwise specified.

### RNA isolation, cDNA synthesis and qPCR

Cellular RNA was extracted with the NucleoSpin RNA II system (Macherey Nagel, Düren, Germany) and reverse transcribed with the iScript cDNA synthesis kit (BIO-RAD, Helsinki, Finland). qPCR was done with TaqMan Gene Expression Assays (Applied Biosystems, Stockholm, Sweden) for *Actb* (Mm00607939_s1), *Arg1* (Mm00475988_m1), *Mrc1* (Mm00485148_m1), *Nos2* (Mm00440485_m1) and *Nt5e* (Mm00501915_m1) for mouse samples. For human samples, *MRC1* (Hs00267207_m1), *B2M* (Hs00984230_m1), *NT5E* (Hs00159686_m1) and *CCL19* (Hs00171149_m1) were used. qPCR was done on a 7900HT Fast Sequence Detection System (Applied Biosystems, Stockholm Sweden). Expression levels for the mouse samples were calculated with the 2^(-ddCT) method using *Arg1* as control [31].

### Cytokine measurements

Mouse peritoneal lavage fluids were analyzed with a Bio-Plex Pro Mouse 23-plex assays, human cell culture supernatants with Bio-Plex Pro Human Cytokine 21-plex and 27-plex assays (BIO-RAD, Helsinki, Finland). In both cases, samples were centrifuged and cell-free supernatants were used for the assays according to the manufacturer’s instructions.

### Inhibition of ecto-5’-nucleotidase activity with AMPCP

α,β-Methyleneadenosine 5′-diphosphate (AMPCP, also known as APCP or AOPCP) is a specific inhibitor of the enzymatic activity of CD73 [32]. To confirm the inhibitory activity of AMPCP, 50000 human PBMCs (containing CD73-positive cell types) were either treated with 100 μM AMPCP (Sigma, M8386, Helsinki, Finland) for 25 minutes or left untreated before measuring the ecto-5’-nucelotidase activity by incubating for 1 hour with 300 μM [2-^3^H]AMP. The role of CD73 activity in monocyte polarization was then studied by pre-incubating purified human monocytes with or without 100 μM AMPCP for 25 minutes, before polarizing them for 3 days (as described above) in the presence or absence of 100 μM AMPCP.

### Statistical analyses

Data were analyzed using a two-tailed Mann-Whitney test with GraphPad Prism 6.02 (GraphPad Software, San Diego, USA). Values of *P* < 0.05 were considered as statistically significant.

## Results

### CD73 is not induced in polarized, anti-inflammatory monocytes/macrophages in humans

**Fig 1:**
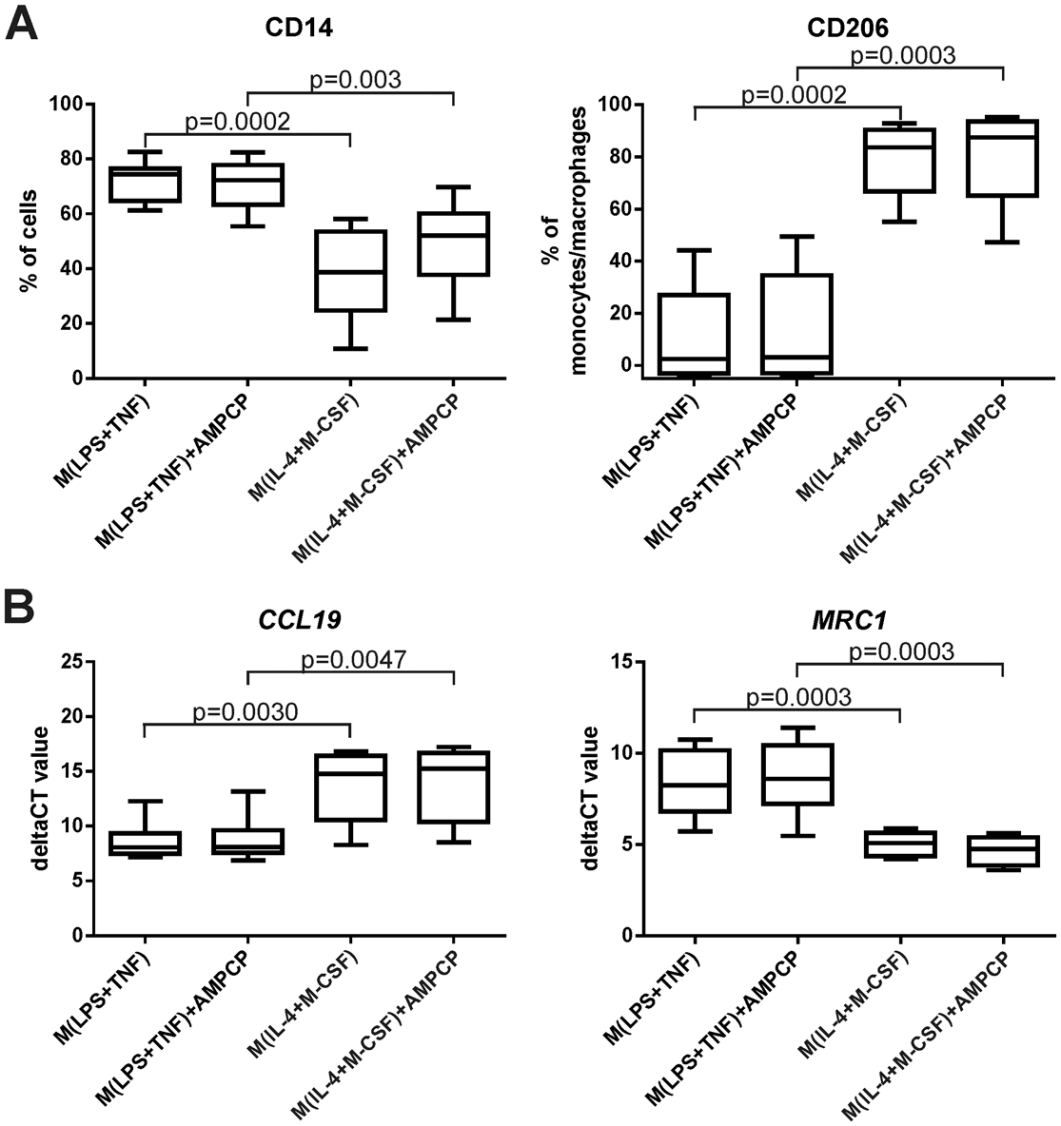
CD73 activity is dispensable for polarization of human monocytes/macrophages. Purified human monocytes were polarized with LPS+TNF or IL-4+M-CSF in the presence or absence of CD73 inhibitor AMPCP for 3 days. **(A)** Flow cytometric analyses of CD14 and CD206 surface expression. **(B)** qPCR analyses of *CCL19* and *MRC1* expression. Results are shown as boxplots with 5–95 percentiles (n = 8 different donors) from 4 different experiments. None of the differences between the AMPCP-treated and control cells were statistically significant under any condition.

To assess the expression level of CD73 in polarized human macrophages, monocytes were isolated from blood and cultured *in vitro* with TNF and LPS stimulation to induce pro-inflammatory M(LPS+TNF) cells or with IL-4 and M-CSF to induce anti-inflammatory M(IL-4+M-CSF) cells. The successful polarizations were confirmed by comparing the expression of standard phenotypic polarization markers between M(LPS+TNF) and M(IL-4+M-CSF) cells. The results showed an increase in the expression of the pro-inflammatory gene *CCL19* (lower CT values), secretion of high levels of pro-inflammatory cytokines such as IL-6, IL-12 and IFN-γ and induction of CD14 in M(LPS+TNF) polarized cells when compared to M(IL-4+M-CSF)-polarized cells (Fig 1 and S2 Fig). Polarization with LPS alone (M(LPS)) or in combination with IFN-γ (M(LPS+IFN-γ)) showed in general a similar polarization profile as obtained with the combination of LPS and TNF. However, the expression of the M2 markers CD206 and MRC1 as well as the levels of CD14 appeared even lower on M(LPS+IFN-γ) cells (S3 Fig). Polarization with IL-4 and M-CSF, in contrast, induced the prototype M2 marker macrophage mannose receptor at the mRNA (*MRC1*) and protein (CD206) level (Fig 1).

**Fig 2:**
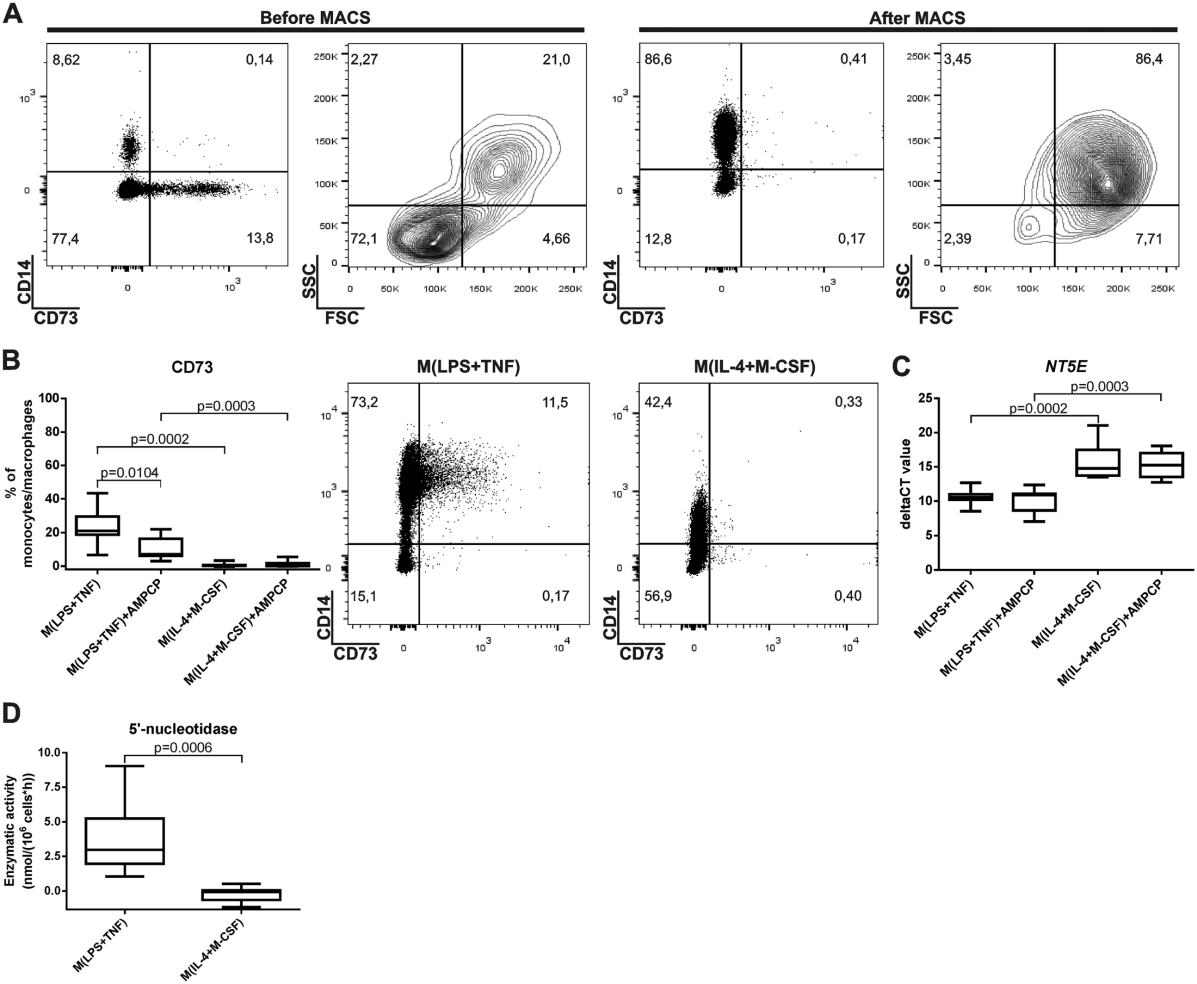
Induction of CD73 on M(LPS+TNF)-polarized human macrophages. **(A)** The expression of cell-surface CD73 on peripheral blood mononuclear cells and macrophages and the purity of the macrophage population after MACS-selection is shown. Ficoll-isolated cells were analyzed by flow cytometry before and immediately after MACS selection for CD14 (a monocyte marker) and CD73 expression and for forward (FSC) and side (SSC) scatters. The percentages of cells falling into different quadrants are indicated. **(B)** The average CD73 expression on CD14^+^ monocytes polarized with and without 100 μM AMPCP to M(LPS+TNF) or M(IL-4+M-CSF) cells after 3 days. Box plots and representative dot plots for each polarization are shown. **(C)** Expression of *NT5E* (= CD73) on the mRNA level after the indicated polarizations is shown as deltaCT values. **(D)** Enzymatic activity of 5’-ectonucleotidase (= CD73) after the indicated polarization. In (B), (C) and (D) the vertical line represents the median and the whiskers the 5–95 percentiles. Pooled data (n = 7–8 different donors) from at least 2 different experiments are shown.

On average, the purity of the monocytes following MACS selection was >87%, as determined by CD14 positivity, and no CD73 expression was detected on these cells (Fig 2A). After monocyte/macrophage polarization for 3 days, cells were gated as shown in S1A Fig. Analysis of CD73 protein expression by flow cytometry showed no expression in M(IL-4+M-CSF) cells and a slight up-regulation after M(LPS+TNF) induction (Fig 2B). Faint CD73 protein induction was also seen when LPS was used alone (M(LPS)) or in combination with IFN-γ (M(LPS+IFN-γ)) to polarize the cells (S3 Fig). Significantly higher *NT5E* (= CD73) gene expression (shown by lower CT values) was found in M(LPS+TNF) cells compared to the M(IL-4+M-CSF) cell type (*P* = 0.0003) (Fig 2C). A consistent and significant increase of ecto-5’-nucleotidase (CD73) enzyme activity was also detected in M(LPS+TNF) cells compared to M(IL-4+M-CSF) cells (*P* = 0.0006) (Fig 2D). In analyses of other purinergic enzyme activities, we found no detectable difference in ATPase activity of the two polarized monocyte/macrophage populations. However, there was a significant decrease in ADPase activity in M(LPS+TNF) cells in comparison to M(IL-4+M-CSF)-polarized cells (S4 Fig). Thus, in human monocytes/macrophages CD73 protein and enzyme activity are only detected after polarization to M(LPS+TNF) cells but not after polarization to anti-inflammatory M(IL-4+M-CSF) cells.

### The polarization of human monocytes/macrophages is not affected by the enzymatic activity of CD73

After verifying the efficiency of the CD73 inhibitor AMPCP (S5 Fig), we determined if the enzymatic activity of CD73 has an effect on the polarization of human monocytes/macrophages. After a 25 minute pre-incubation with 100 μM AMPCP, human monocytes were cultured and polarized for 3 days in the presence of 100 μM AMPCP. Respective control cells were not subjected to AMPCP at any step. The inhibition of the enzymatic activity of CD73 slightly reduced surface levels of CD73, although it did not affect CD73 mRNA expression. Notably, when the induction of pro-inflammatory (CCL19 mRNA) and anti-inflammatory (MRC1 mRNA and protein) markers were compared, no differences were found between AMPCP-inhibited and control cells in either M(LPS+TNF) or M(IL-4+M-CSF) polarized cells (Fig 1). Furthermore, we tested if the addition of AMP, the substrate of CD73, has an effect on the polarization of human monocytes/macrophages. Purified human monocytes were therefore cultured and polarized to M(LPS+TNF) or M(IL-4+M-CSF) cells in the absence or presence of 10 μM AMP. The results showed that addition of exogenous AMP did not significantly affect the expression of CD73 or any of the polarization markers (S7 Fig). Thus, CD73 activity in monocytes/macrophages is fully dispensable for their polarization.

### CD73 is present in resting, but absent in elicited pro-inflammatory M(LPS) or anti-inflammatory M(IL-4c) polarized peritoneal mouse macrophages *in vivo*

To assess the induction of CD73 during macrophage polarization *in vivo*, we utilized wild-type and CD73-deficient mice. First, inflammation and macrophage proliferation was elicited by i.p. injection of thioglycollate. After this, cell-differentiation was induced by i.p. administered LPS to a pro-inflammatory M(LPS) direction or by i.p. administered IL-4-anti-IL-4 complex to an anti-inflammatory M(IL-4c) direction.

**Fig 3:**
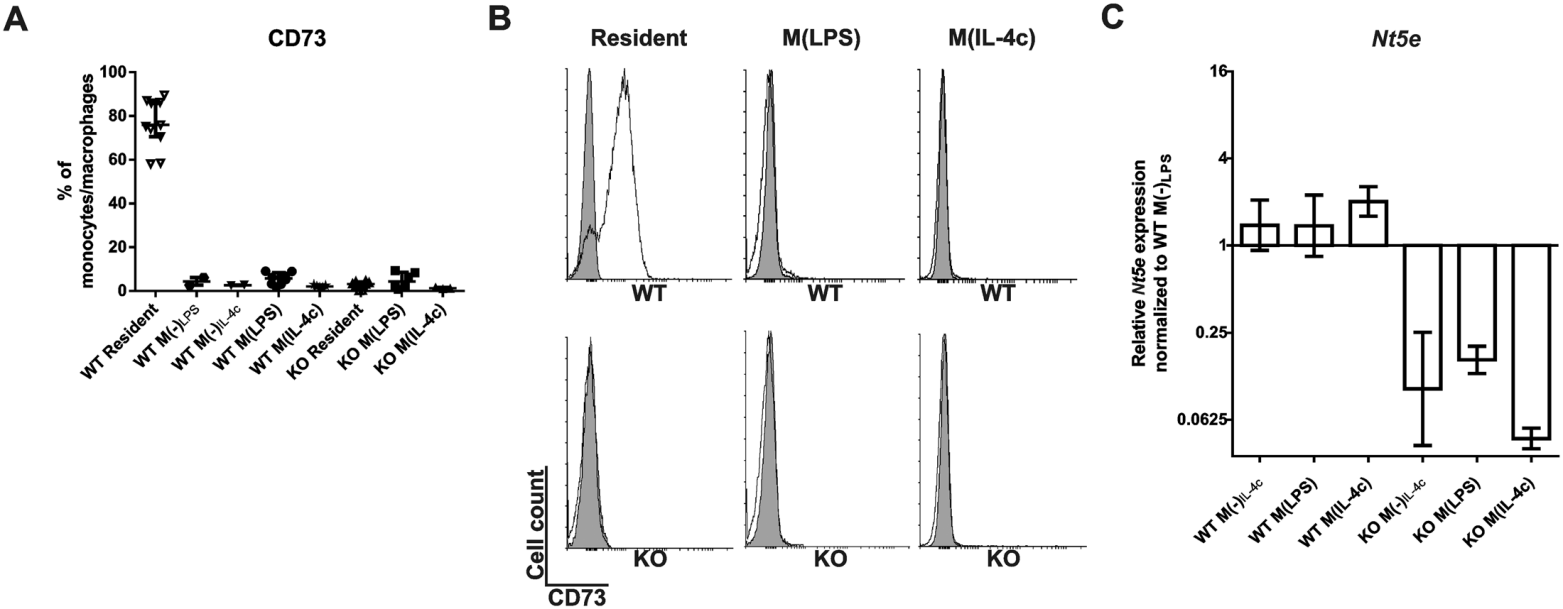
Elicited and polarized murine peritoneal macrophages are CD73 negative. The *in vivo* polarization was induced by i.p. thioglycollate + LPS (M(LPS)) and thioglycollate plus IL-4-anti-IL-4 antibody complex (M(IL-4c)) in wild-type and CD73-deficient mice. In control mice, thioglycollate only was injected for 16 h followed by PBS for 6 h (M(-)_LPS_) or thioglycollate only was injected for 2 d followed by PBS for another 2 d (M(-)_IL-4c_). **(A)** The percentage of peritoneal macrophages expressing CD73 in WT and KO animals is shown after different treatments (6–8 mice/group (except in M(-) control groups, where n = 2–3); data are from at least 5 different experiments). **(B)** Representative histograms (specific stainings in white, isotype control stainings in grey) depict the expression levels of CD73 on resident, M(LPS) and M(IL-4c) wild-type and KO mice. **(C)** *Nt5e* (= CD73) mRNA expression levels in the indicated peritoneal macrophage populations. Expression levels relative to WT M(-)_LPS_ were calculated with the ddCT method using beta actin as a control gene. The relative gene expression is shown (mean with SEM (*n* = 8) from 4 different experiments).

**Fig 4:**
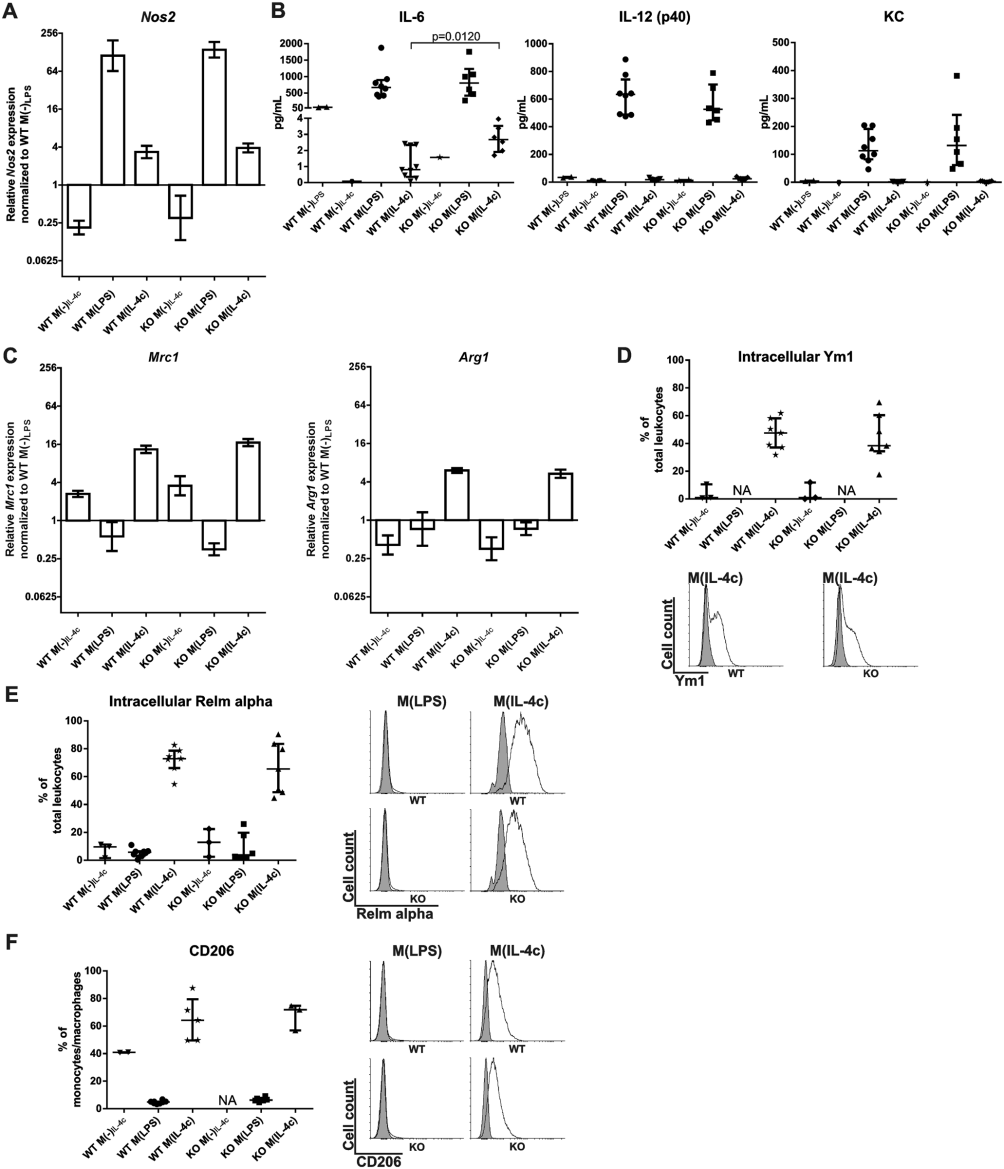
Normal macrophage polarization in CD73-deficient animals. **(A)** Gene expression (mean ± SEM) of *Nos2* (a pro-inflammatory macrophage marker) in peritoneal cells after the indicated *in vivo* polarization was determined using qPCR. **(B)** The concentrations of the cytokines IL-6, IL-12 (p40) and KC (pro-inflammatory macrophage markers) in the peritoneal lavage fluid of wild-type (WT) and CD73-deficient (KO) mice after the *in vivo* polarizations. **(C)** Gene expression (mean ± SEM) of *Mrc1* and *Arg1* (both anti-inflammatory macrophage marker) in peritoneal cells after *in vivo* polarization was determined using qPCR. **(D)** Intracellular expression of Ym-1, **(E)** intracellular expression of Relm alpha and (**F**) surface expression of CD206 (all anti-inflammatory macrophage markers) was determined on total peritoneal leukocytes using flow cytometry and is shown together with representative histograms (specific stainings in white, isotype control stainings in grey). All data are from 6–9 mice/group (except in M(-) control groups where *n* = 2–4 and in F where *n* = 3–8). In (B, D, E, F) each dot represents an individual experimental value, the vertical line the median and the whiskers the interquartile ranges. Data are from at least 4 different experiments. NA = not available.

When resident peritoneal macrophages from wild-type mice were analyzed for CD73 surface expression, virtually all cells were CD73 positive (Fig 3A and 3B). Absence of anti-CD73 antibody reactivity with macrophages isolated from CD73 deficient mice confirmed the specificity of the stainings. Strikingly, elicited peritoneal macrophages did not show any expression of CD73 in wild-type mice. This did not change following either M(LPS) or M(IL-4c) polarization (Fig 3A and 3B). In addition, the very low expression levels of the *Nt5e* gene in peritoneal leukocytes were similar after both polarization protocols in wild-type mice (Fig 3C). Induction of *Nos2 and* secretion of pro-inflammatory cytokines such as IL-6, IL-12 and KC into the peritoneum verified a successful polarization after M(LPS) stimulation, while M(IL-4c) polarization was verified by induction of *Mrc1* and positivity for Ym1, Relm alpha and anti-CD206 (Fig 4). Thus, after *in vivo* polarization of mouse peritoneal macrophages to either M(LPS) or M(IL-4c) direction no CD73 is detectable on these cells.

### Normal *in vivo* polarization of elicited peritoneal macrophages in CD73 deficient mice

When comparing the polarization of peritoneal macrophages in wild-type and CD73^-/-^ animals, we found similar inductions of *Nos2* in M(LPS) and of *Mrc1* and *Arg1* in M(IL-4c)-cells in both genotypes (Fig 4A and 4C). Furthermore, flow cytometric stainings revealed that peritoneal leukocyte populations in wild-type and CD73^-/-^ mice did not differ in their percentage of cells positive for Ym1 (intracellular), Relm alpha (intracellular) or CD206 (surface) (Fig 4D–4F). Collectively these data show that CD73 activity from Treg, or from any other leukocyte or non-hematopoietic cell type, is fully dispensable for polarization of peritoneal macrophages *in vivo*.

**Fig 5:**
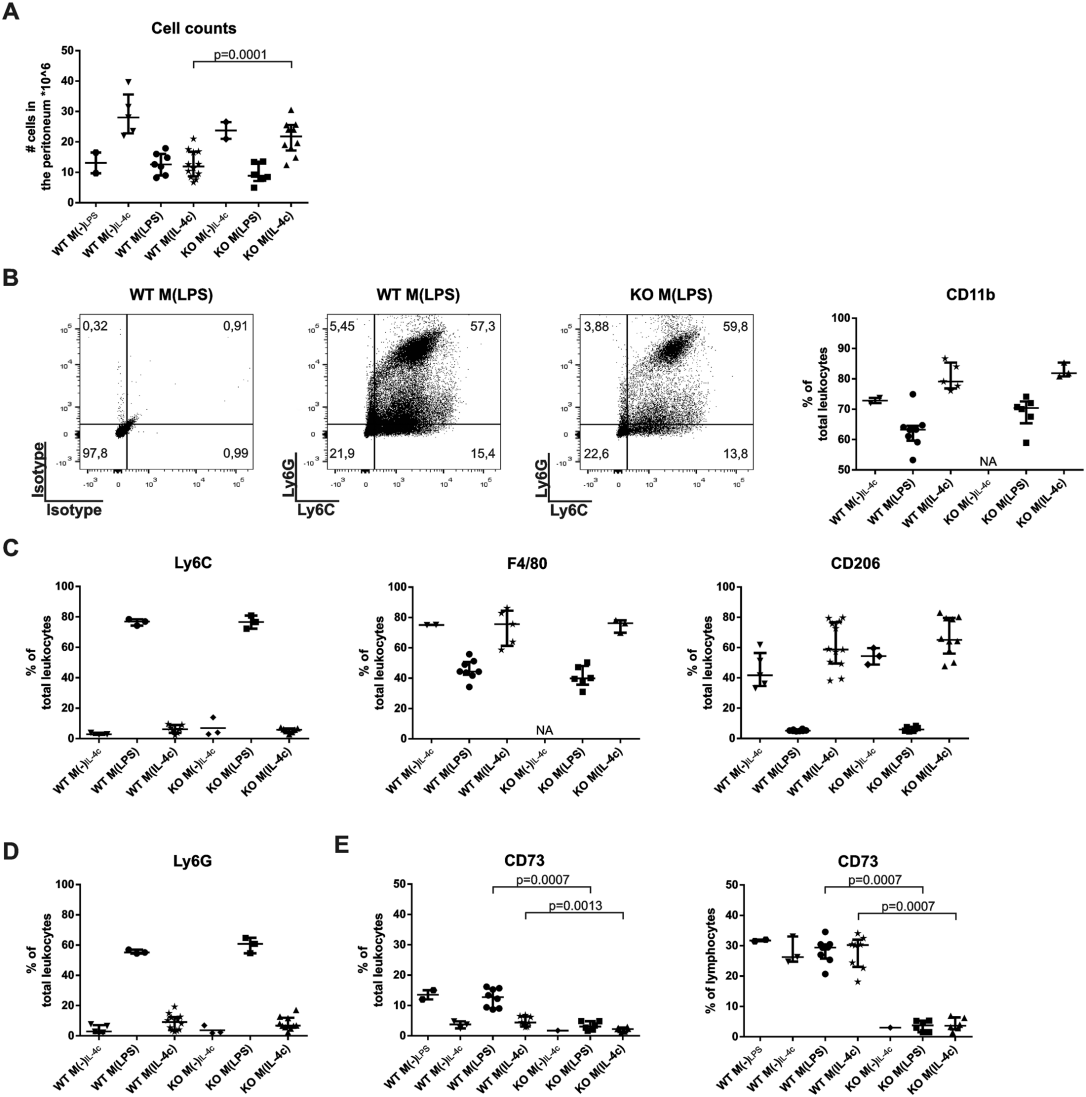
Normal leukocyte infiltration to the peritoneum in CD73-deficient mice after M(IL-4c) inflammation. **(A)** Total leukocyte counts (median with interquartile ranges) in the peritoneal fluid after the indicated polarizations. **(B-D)** Phenotype of peritoneal myeloid cells after the indicated *in vivo* polarizations. Ly6C and F4/80 are macrophage markers, CD206 an anti-inflammatory macrophage marker, Ly6G, a granulocyte marker and CD11b a myeloid marker. The percentages of total peritoneal leukocytes positive for each marker are shown with representative stainings. **(E-F)** CD73 expression on (**E**) total peritoneal leukocytes and (**F**) on peritoneal lymphocytes. Each dot represents an individual experimental value, the vertical line the median and the whiskers the interquartile ranges. Data are from at least 3 different experiments. NA = not available.

In CD73 deficient mice, the total numbers of leukocytes migrating to the peritoneal cavity were similar to those in wild-type mice after M(LPS)-polarization, and slightly increased following M(IL-4c) polarization (Fig 5A). However, there was no difference in the percentages of peritoneal myeloid cells (double positive for Ly6C and Ly6G or expressing CD11b), macrophages (as defined by Ly6C, F4/80 or CD206 positivity) or granulocytes (Ly6G-positive) between wild-type and CD73-deficient mice when compared either after M(LPS) or M(Il-4c) inductions (Fig 5B–5D). Moreover, the levels of cytokines secreted to the peritoneal fluid during macrophage polarization were very similar in wild-type and CD73^-/-^ mice (S6 Fig). Differences following M(LPS) polarization could only be found in animals deficient for CD73 for RANTES, which showed reduced levels, or after M(IL-4c) polarization in G-CSF, IL-3, IL-6, MCP-1, MIP-1b and RANTES, which showed slightly enhanced levels (Fig 4 and S6 Fig). Finally we noticed that while M(LPS) or M(IL-4c) stimuli did not affect the percentage of lymphocytes (about 30% of lymphocytes were positive under both conditions) expressing CD73 in wild-type mice, M(LPS) polarized animals nevertheless showed higher CD73 expression within the total peritoneal leukocyte pool (Fig 5E). Based on our FACS analyses these CD73 positive cells were granulocytes. Thus, CD73 activity appears to have modest effects on the numbers of infiltrating cells in the inflamed peritoneum. In striking contrast, although there are CD73-positive macrophages present in the resting peritoneal cavity, and CD73-positive lymphocytes (and granulocytes) under M(LPS) and M(IL-4c) polarization conditions, CD73 activity from these hematopoietic (or other cells) has no detectable effect on peritoneal macrophage polarization.

## Discussion

We report here that polarization of mouse peritoneal macrophages (M(LPS) vs. M(IL-4c)) *in vivo* as well as polarization of human blood-derived monocyte/macrophages (M(LPS+TNF) vs (M(IL-4+M-CSF)) towards either a pro-inflammatory, or an anti-inflammatory, phenotype is a completely CD73 independent process. In addition, only human *in vitro* polarized pro-inflammatory M(LPS+TNF) (as well as M(LPS) and M(LPS+IFN-γ)) macrophages show a small detectable expression of CD73, while other polarized human or mouse macrophages do not. Our findings are intriguing, since exogenous adenosine is reported to drive cultured peritoneal macrophages to an anti-inflammatory phenotype [22,27]. Furthermore, on lymphocytes CD73-derived adenosine is functionally critical for immunosuppressive functions of Tregs [1,2,6], which includes guiding macrophage differentiation towards an anti-inflammatory phenotype [8,9].

While we did not detect any CD73 expression on freshly isolated CD14^+^ monocytes, about 5% of a specific CD14^+^CD16^+^ subpopulation, representing ~2–10% of all blood monocytes, has been reported to express CD73 [24]. This CD14^+^CD16^+^ population of monocytes is increased in some disease conditions and is considered to be pro-inflammatory, therefore being in line with our findings of CD73 expression on M(LPS+TNF) cells [24,33].

The expression of human CD73 during macrophage differentiation has not been analyzed earlier. Our finding of an increased AMPase (CD73) activity in human M(LPS+TNF) macrophages in combination with lowered ADPase activity suggests that in these cells more ADP accumulates while at the same time more AMP is converted to adenosine. Significant upregulation of CD73 mRNA and protein expression in M(LPS+TNF) polarized cells support this notion. Noteworthy, also the polarization with LPS alone to M(LPS) and with LPS and IFN-γ to M(LPS+IFN-γ) lead to a detectable expression of CD73 on those cells. Thus, in contrast to previously published mouse data [12,27], in human monocytes/macrophages the expression and activity of CD73 was on M(LPS+TNF) cells and was not detectable on M(IL-4+M-CSF)-polarized cells. However, the very low levels of CD73 on human blood monocytes/macrophages had no effect on their polarization to either a pro-inflammatory M(LPS+TNF) or an anti-inflammatory M(IL-4+M-CSF) direction since the inhibition of CD73 activity with AMPCP did not alter this process. It has been shown earlier that high adenosine levels can influence the polarization behavior of macrophages [5,21,34], however, our results indicate that this effect is not CD73 dependent.

In contrast to the human cells, we did not see any surface expression of CD73 in mouse peritoneal monocytes/macrophages after polarization to either M(LPS) or M(IL-4c) cells. In addition, the very low expression levels of the *Nt5e* gene in peritoneal leukocytes were similar after both polarization protocols in wild-type animals. Thus, *in vivo* elicited mouse peritoneal cavity macrophages do not upregulate CD73 following their polarization. Injection of pro-inflammatory mediators into the peritoneum causes the resident macrophages to vanish from the peritoneum and the replacement of these cells with newly arrived blood-derived monocytes, which will mature into inflammatory peritoneal macrophages [35]. This is especially noteworthy, as recent fate mapping experiments have shown that resident peritoneal macrophages are derived from yolk sac and therefore are of different origin than elicited macrophages [14,15,36]. We thus believe that in our experiments the resident CD73-positive peritoneal macrophages are replaced by CD73-negative blood monocytes, which, at least after the polarization protocols we used, do not induce CD73. This would also readily explain the discrepancy in results on CD73 expression in murine macrophages between us and two previous studies [12,27], both of which used resident peritoneal macrophages. Thus, in those studies polarization of resident, constitutively CD73-positive macrophages were analyzed under *in vitro* conditions, in which the impact of monocyte/macrophage dynamics was ignored. Therefore, we believe that our data firmly show that under physiologically relevant conditions CD73 is not induced in M(IL-4c) cells in the inflamed peritoneal cavity.

Overall, there was almost no difference in any of the polarization markers between the wild-type and the CD73 deficient animals after either M(LPS) or M(IL-4c)-polarization. This is strong genetic evidence that the presence or absence of host CD73 activity in any cell type does not play a significant role for peritoneal macrophage polarization *in vivo*, despite the reported polarizing effect of adenosine on macrophages [5,21,22]. These results are intriguing, especially since Tregs, whose immunosuppressive functions are thought to be CD73-dependent, are reported to be instrumental in guiding the polarization of anti-inflammatory macrophages *in vitro* [7–9]. Nevertheless, the cytokines G-CSF, IL-3, IL-6, MCP-1, MIP-1b and RANTES showed slight differences between the genotypes. While most of these differences are likely caused by the different cell numbers following M(IL-4c) polarization, RANTES (CCL5) showed decreased levels in the M(LPS) polarized CD73-deficient animals. RANTES has been described as a cytokine which can be involved in macrophage activation and pro-inflammatory macrophage polarization [37]. Hence, lower levels could be suggestive of a diminished polarization towards a pro-inflammatory phenotype, even though none of our other data would support this. The absence of CD73 only slightly affected the cytokine levels in our experimental setup, however, in a severe sepsis model it did cause an increase in the pro-inflammatory cytokines [34]. Collectively, our data thus show that CD73-derived adenosine production from any cell type is not needed for M(IL-4c)-polarization in the peritoneum *in vivo*.

As macrophage polarization *in vivo* is highly dynamic, it was crucial to establish polarization regimens that produce the expected macrophage phenotypes. Therefore, we verified the polarization status of the populations (more than 87% CD14-positive monocytes at the beginning of the culturing) by analyzing the expression of several specific surface molecules, genes as well as cytokines. Our finding that human pro-inflammatory M(LPS+TNF) cells expressed a significantly higher percentage of CD14^+^ cells in comparison to anti-inflammatory M(IL-4+M-CSF)-polarized cells is in line with other TNF-based polarization models [38]. Furthermore, high levels of the gene *CCL19* as well as pro-inflammatory cytokines such as IL-6, IL-12 and IFN-γ in these cells are characteristic for pro-inflammatory macrophages [13–18,38–43]. On the other hand, induction of CD206 is a hall mark of anti-inflammatory macrophage polarization [44–46]. In the mouse models, we used thioglycollate to elicit inflammation and macrophage proliferation. This was followed by LPS to promote pro-inflammatory M(LPS) differentiation [44,45] or by IL-4-anti-IL-4 complex to promote anti-inflammatory M(IL-4c) differentiation [30]. Upregulation of the gene *Nos2* and of cytokines such as IL-6 and IL-12 in M(LPS) mouse macrophages, and induction of canonical anti-inflammatory macrophage markers CD206, Ym1 and Relm alpha in M(IL-4c) cells were consistent with the expected polarization patterns *in vivo*. Notably, longer than one-day polarization with thioglycollate and LPS invariably led to the induction of anti-inflammatory-type cells, which is in line with published reports [46]. It was also critical to our conclusions that we carefully controlled the effects of non-specific antibody binding to the multiple Fc-receptors expressed on macrophages. Nevertheless, at least two clear limitations should be kept in mind when interpreting our results. First, the origin and polarization of macrophages was different in human (blood monocyte-derived, *in vitro* polarized with TNF+LPS/IL-4+M-CSF) and mouse (activated peritoneal macrophages, *in vivo* polarized with LPS vs IL-4+IL-4-antibody), which may explain the differential CD73 expression patterns in the two species. Moreover, the developmental origin and differentiation of tissue-resident and monocyte-derived macrophages varies widely between different tissues. Thus, it remains fully possible that the induction and role of CD73 in macrophage polarization might be different in some other tissue.

In conclusion, in our experimental models the induction of macrophage CD73 differs between human and mouse, the elicited mouse peritoneal macrophages do not express CD73 upon *in vivo* polarization, and CD73 activity from any host cell type is not required for the monocyte/macrophage polarization in the peritoneum towards a pro- or an anti-inflammatory phenotype *in vivo*.

## Supporting Information

S1 Fig Gating strategies for human and mouse monocytes/macrophages. **(A)** The gating of human monocytes/macrophages according to their FSC/SSC profiles and subgating on CD14^+^. **(B)** The gating of mouse peritoneal monocytes/macrophages according to their FSC/SSC profiles and subgating on F4/80^+^ CD11b^+^. Representative flow cytometric analyses under the different polarization protocols are shown.
(TIF)

S2 Fig The phenotype of the polarized human monocytes/macrophages. The pro- and anti-inflammatory polarizations were confirmed after 3 days by determinations of the concentration of soluble pro-inflammatory cytokines in the culture supernatants (shown as pg/mL (median with interquartile ranges, *n* = 11)). Each dot represents an individual experimental value, the vertical line the median and the whiskers the interquartile ranges.
(TIF)

S3 Fig Similar phenotype of M(LPS), M(LPS+IFN-γ) and M(LPS+TNF) polarized human monocytes. **(A)** The expression of CD14, CD206 and CD73 on cultured MACS-selected monocytes was determined by flow cytometry after M(LPS), M(LPS+IFN-γ) and M(LPS+TNF) polarization for 3 days. **(B)** qPCR analyses of *CCL19*, *MRC1* and *NT5E* expression on polarized cells. Note that high deltaCT values indicate low expression levels. Results are shown as boxplots with the whiskers representing the 5–95 percentiles (n = 3–4 different donors).
(TIF)

S4 Fig Nucleotidase activities in polarized human monocytes/macrophages. Ectonucelotidase (and adenylate kinase) activities were determined using enzymatic assays from human MACS-selected macrophages polarized *in vitro* with the indicated stimuli for 3 days. Each dot represents an individual experimental value form 6 different experiments, the vertical line the median and the whiskers the interquartile ranges.
(TIF)

S5 Fig Enzymatic inhibition of CD73 with AMPCP. The inhibition of ecto-5’-nucleotidase activity by 100 μM AMPCP in total PBMC-population. Data from two different experiments are shown as median with interquartile ranges.
(TIF)

S6 Fig Cytokine secretion in polarized mouse macrophages *in vivo*. The concentrations of the indicated cytokines in the peritoneal lavage fluid of wild-type (WT) and CD73-deficient (KO) mice after the *in vivo* M(LPS), M(IL-4c) or M(-) control polarizations were determined using Multiplex arrays. Data are shown as median with interquartile ranges from 6–9 mice/group (except in M(-) control groups, where *n* = 2–4). Data are from at least 5 different experiments. ND = not detectable.
(TIF)

S7 Fig Addition of the CD73 substrate AMP does not change the polarization of human monocytes/macrophages. Purified human monocytes were polarized with LPS+TNF or IL-4+M-CSF in the presence or absence of 10 μM AMP for 3 days. **(A)** Flow cytometric analyzes of CD14, CD206 and CD73 surface expression. **(B)** qPCR analyses of *CCL19*, *MRC1* and *NT5E* (= CD73) expression. Note that high deltaCT values indicate low expression levels. Results are show as boxplots with the whiskers representing the 5–95 percentiles (n = 3–4 different donors). None of the differences between the AMP-treated and control cells were statistically significant under any condition.
(TIF)

S1 Dataset Raw data from the experiments. (XLS)
