# Supplementary figures and images for: CD73 Activity is Dispensable for the Polarization of M2 Macrophages
Source: PLoS One. 2015 Aug 10;10(8):e0134721. doi: 10.1371/journal.pone.0134721 (PMC4530872; doi:10.1371/journal.pone.0134721)

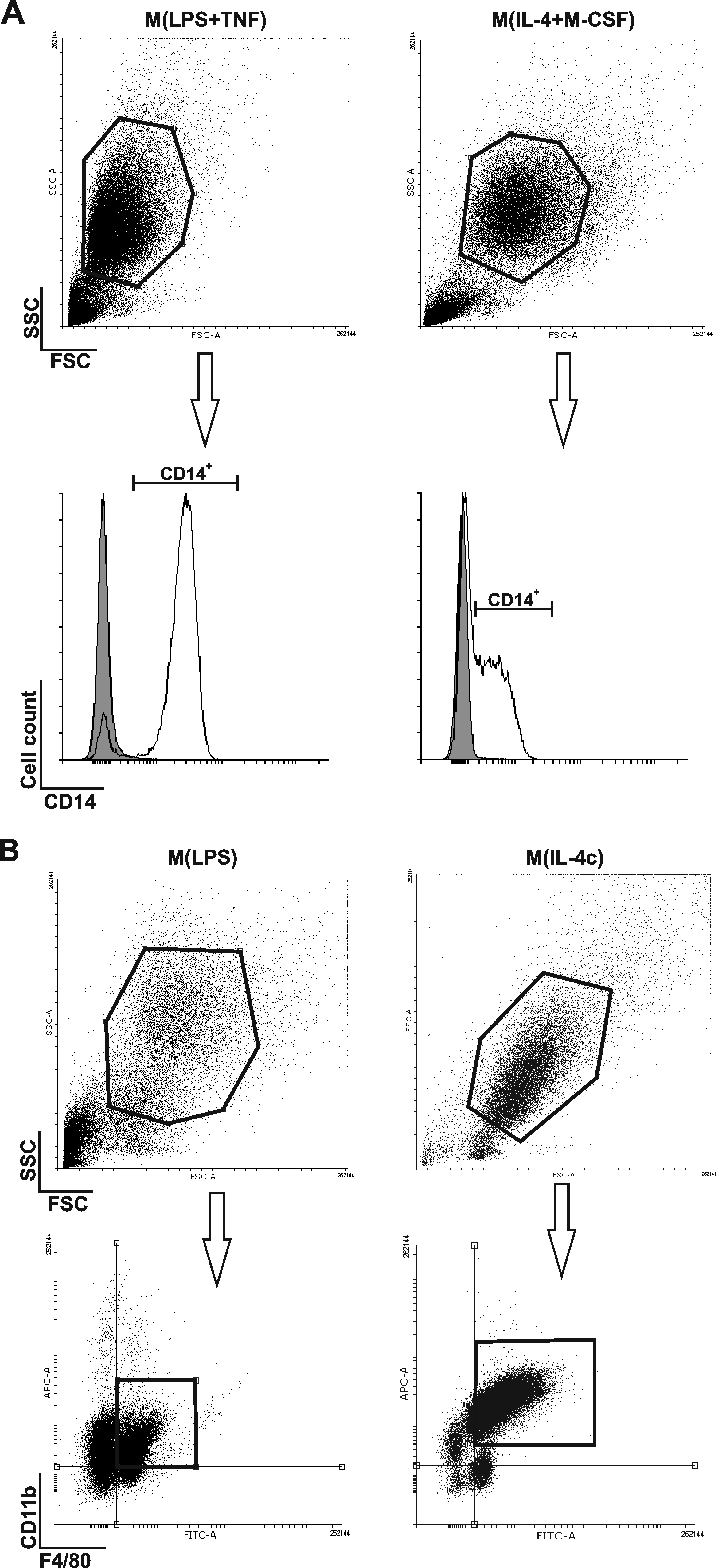

Supplement: S1 Fig — (A) The gating of human monocytes/macrophages according to their FSC/SSC profiles and subgating on CD14+. (B) The gating of mouse peritoneal monocytes/macrophages according to their FSC/SSC profiles and subgating on F4/80+ CD11b+. Representative flow cytometric analyses under the different polarization protocols are shown. (TIF) [file pone.0134721.s001.tif]

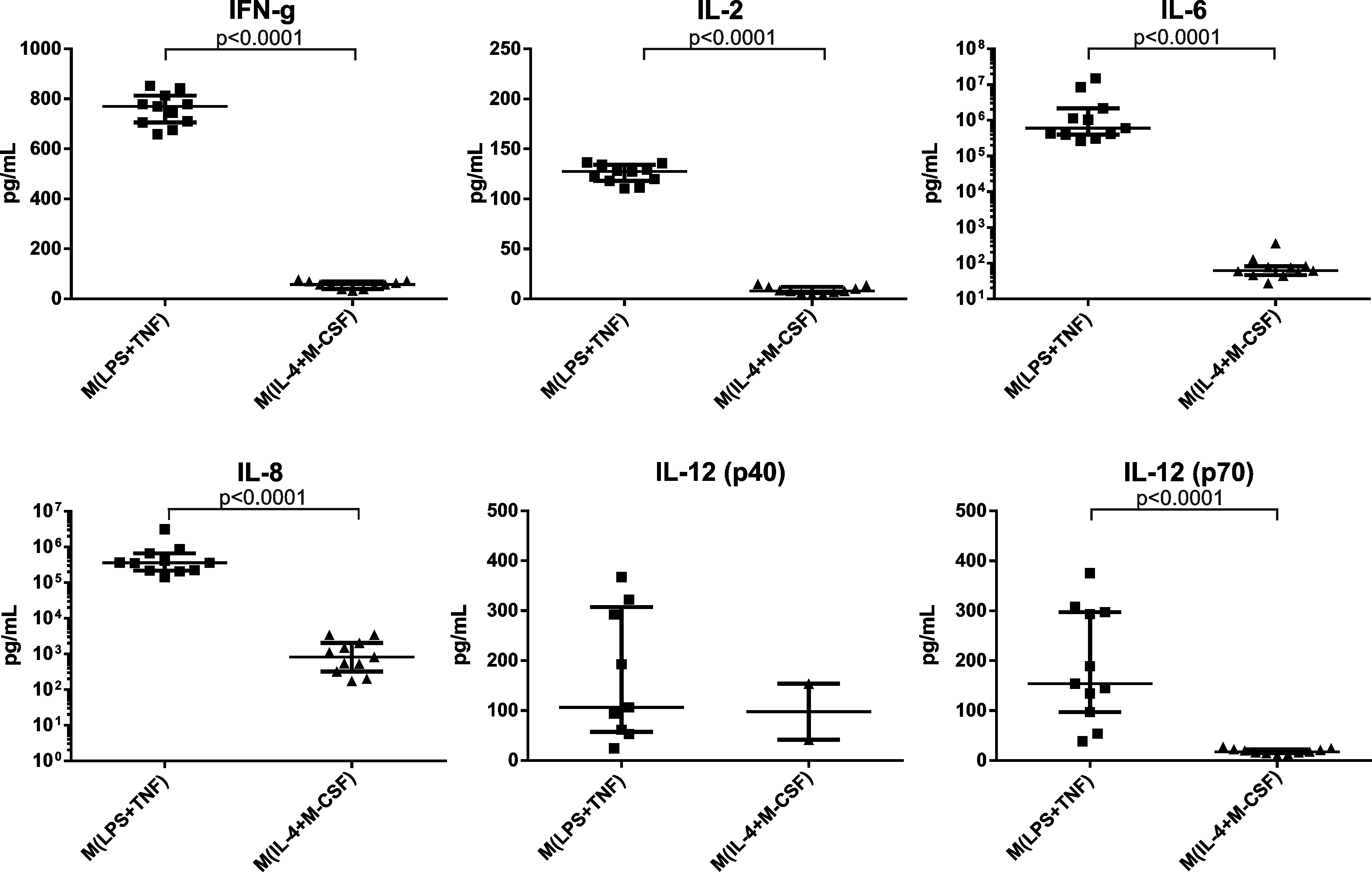

Supplement: S2 Fig — The pro- and anti-inflammatory polarizations were confirmed after 3 days by determinations of the concentration of soluble pro-inflammatory cytokines in the culture supernatants (shown as pg/mL (median with interquartile ranges, n = 11)). Each dot represents an individual experimental value, the vertical line the median and the whiskers the interquartile ranges. (TIF) [file pone.0134721.s002.tif]

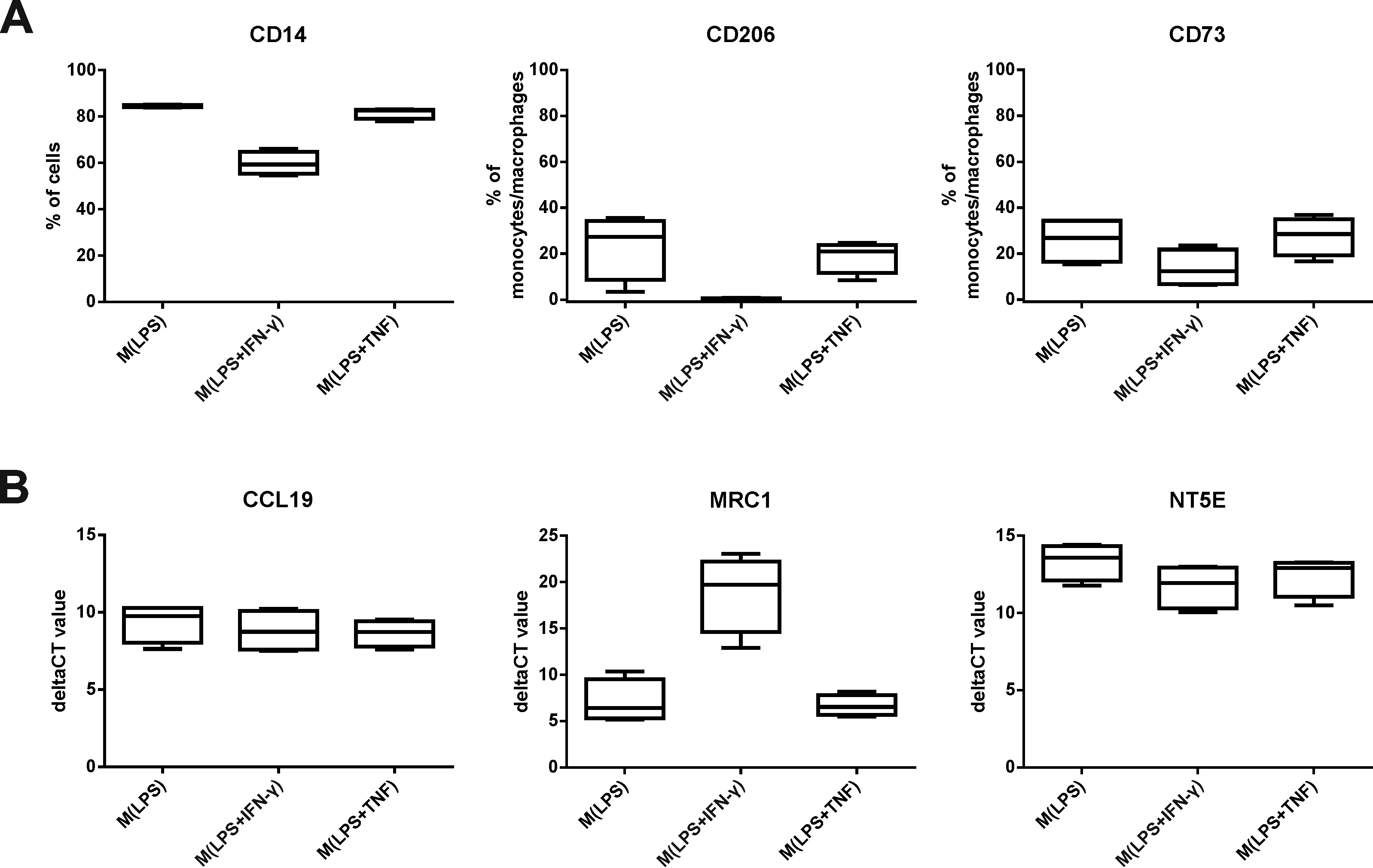

Supplement: S3 Fig — (A) The expression of CD14, CD206 and CD73 on cultured MACS-selected monocytes was determined by flow cytometry after M(LPS), M(LPS+IFN-γ) and M(LPS+TNF) polarization for 3 days. (B) qPCR analyses of CCL19, MRC1 and NT5E expression on polarized cells. Note that high deltaCT values indicate low expression levels. Results are shown as boxplots with the whiskers representing the 5–95 percentiles (n = 3–4 different donors). (TIF) [file pone.0134721.s003.tif]

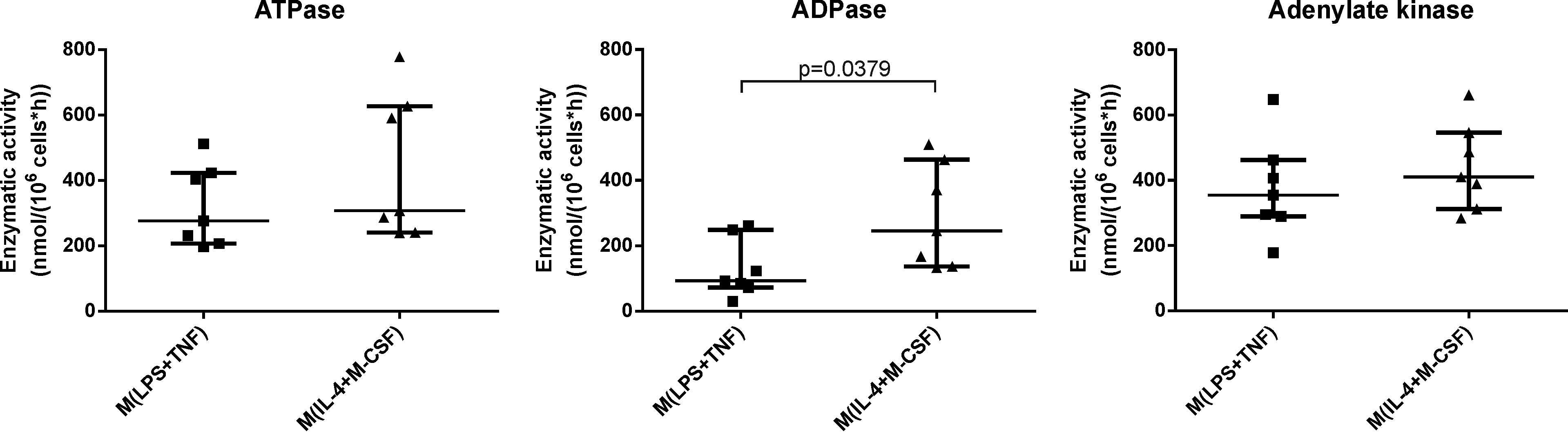

Supplement: S4 Fig — Ectonucelotidase (and adenylate kinase) activities were determined using enzymatic assays from human MACS-selected macrophages polarized in vitro with the indicated stimuli for 3 days. Each dot represents an individual experimental value form 6 different experiments, the vertical line the median and the whiskers the interquartile ranges. (TIF) [file pone.0134721.s004.tif]

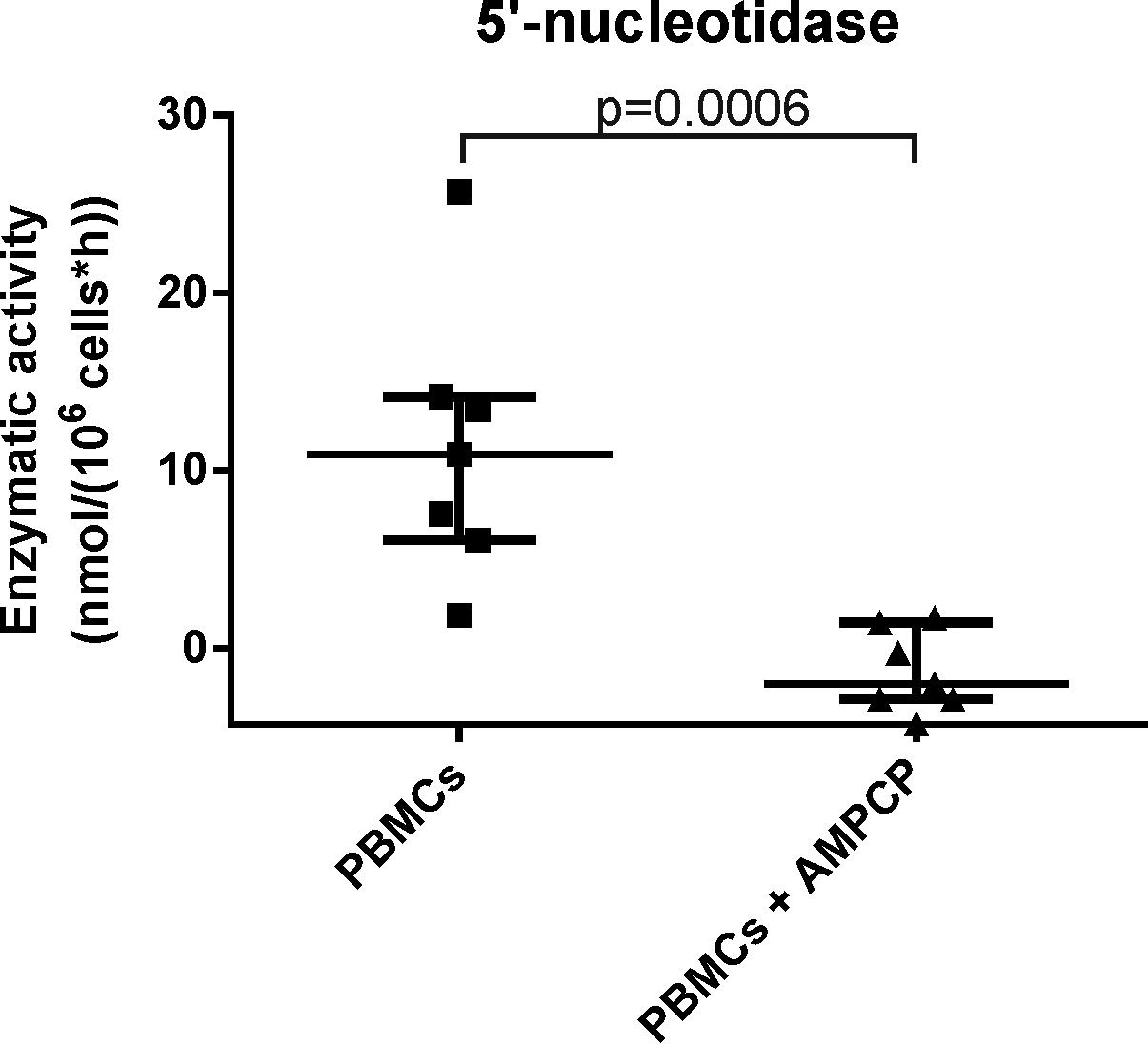

Supplement: S5 Fig — The inhibition of ecto-5’-nucleotidase activity by 100 μM AMPCP in total PBMC-population. Data from two different experiments are shown as median with interquartile ranges. (TIF) [file pone.0134721.s005.tif]

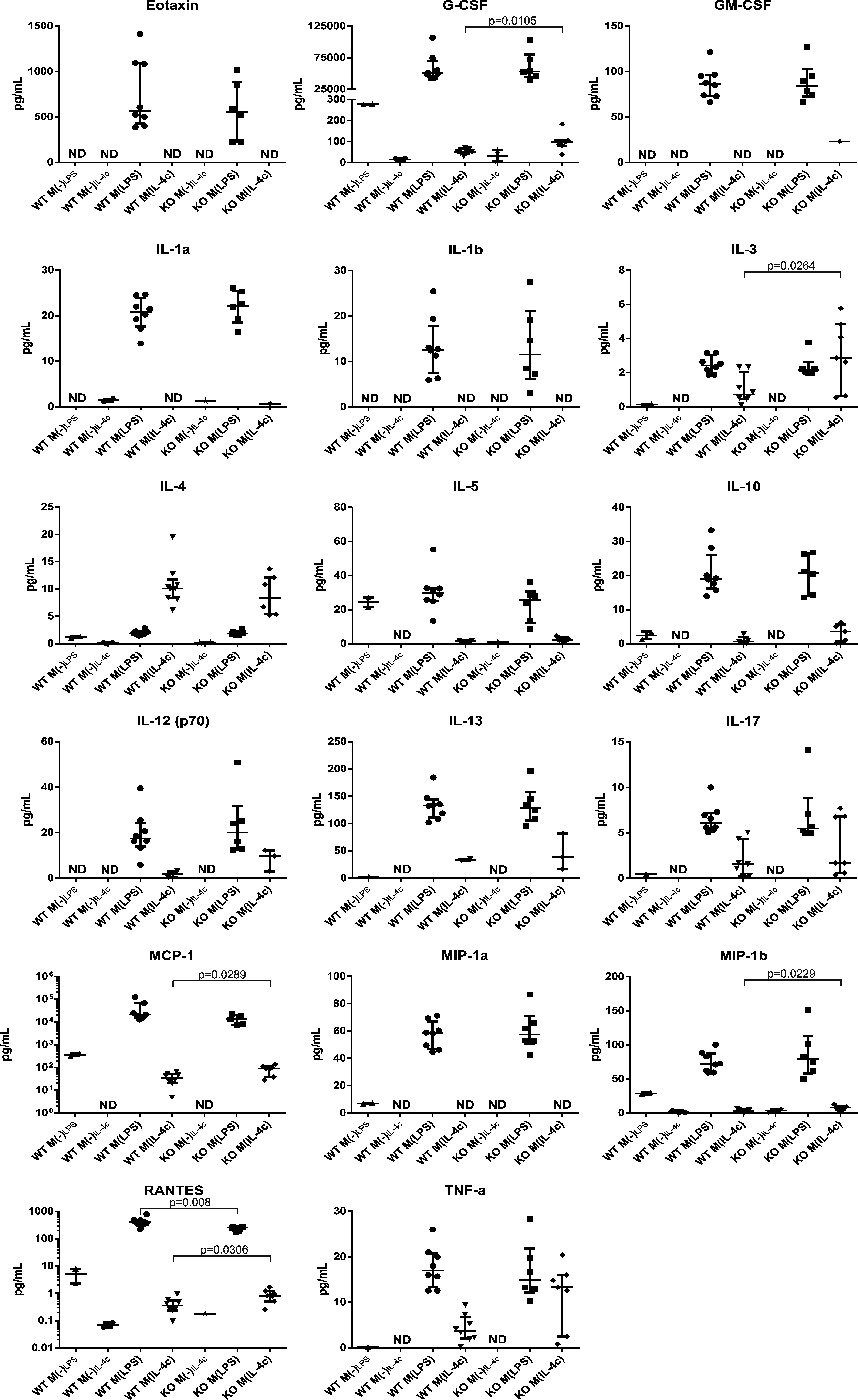

Supplement: S6 Fig — The concentrations of the indicated cytokines in the peritoneal lavage fluid of wild-type (WT) and CD73-deficient (KO) mice after the in vivo M(LPS), M(IL-4c) or M(-) control polarizations were determined using Multiplex arrays. Data are shown as median with interquartile ranges from 6–9 mice/group (except in M(-) control groups, where n = 2–4). Data are from at least 5 different experiments. ND = not detectable. (TIF) [file pone.0134721.s006.tif]

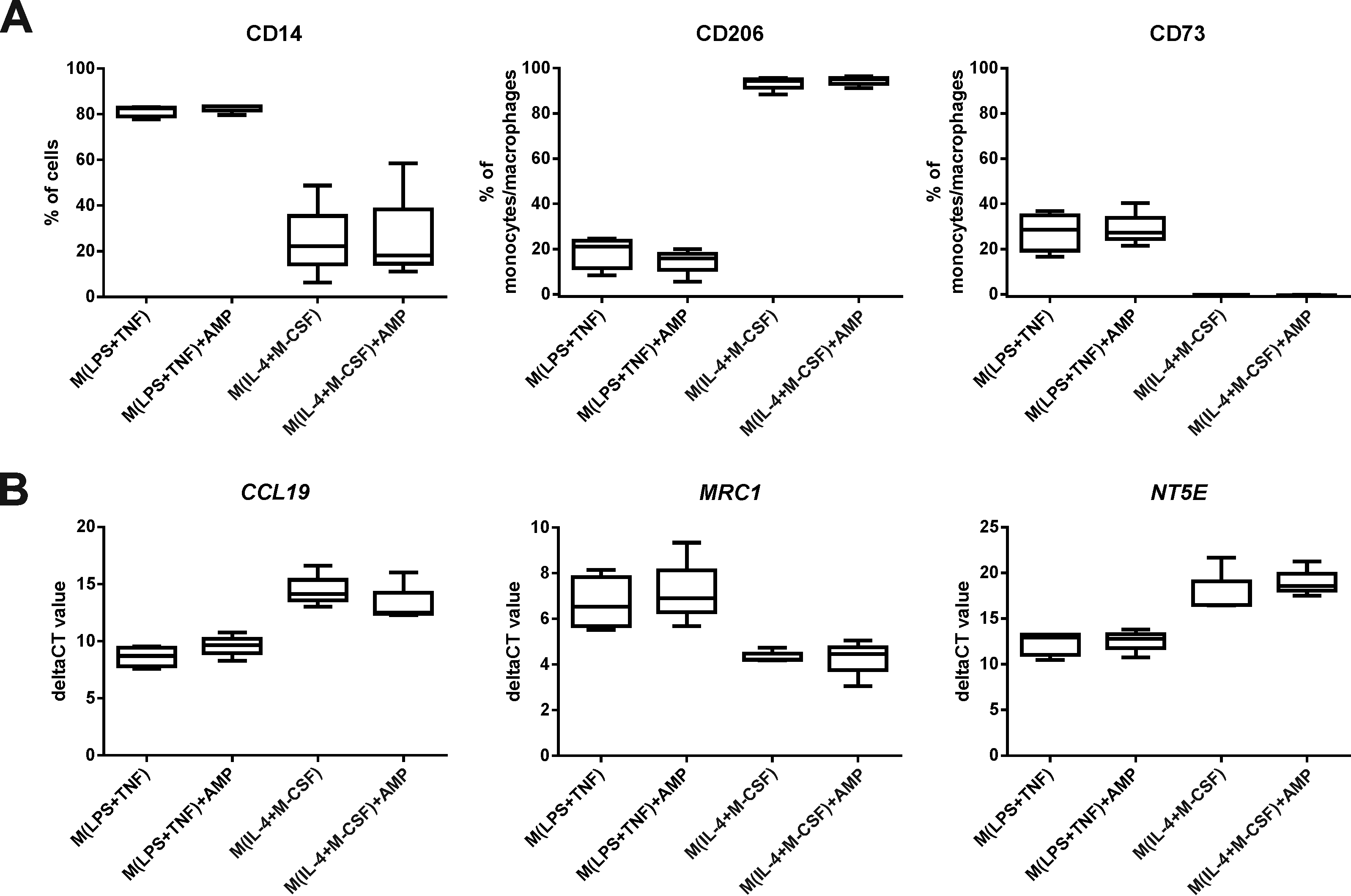

Supplement: S7 Fig — Purified human monocytes were polarized with LPS+TNF or IL-4+M-CSF in the presence or absence of 10 μM AMP for 3 days. (A) Flow cytometric analyzes of CD14, CD206 and CD73 surface expression. (B) qPCR analyses of CCL19, MRC1 and NT5E (= CD73) expression. Note that high deltaCT values indicate low expression levels. Results are show as boxplots with the whiskers representing the 5–95 percentiles (n = 3–4 different donors). None of the differences between the AMP-treated and control cells were statistically significant under any condition. (TIF) [file pone.0134721.s007.tif]
